# Clinical Effect of Uterosacral and Cardinal Ligament Fixation versus Sacrospinous Ligament Fixation of Vaginal Vault Prolapse: A Retrospective Analysis

**DOI:** 10.1155/2023/1489928

**Published:** 2023-06-03

**Authors:** Ling-xiao Huang, Min Guo, Li-xiao Sha, Cong Chen, Xiao-hua Lin, Xiao-xia Dong

**Affiliations:** Department of Gynecology, Wenzhou People's Hospital, Wenzhou 325000, China

## Abstract

**Objective:**

This study aimed at comparing sacrospinous ligament fixation (SSLF) with uterosacral and cardinal ligament fixation (USCLF) concerning complications and outcomes in patients with pelvic organ prolapse (POP).

**Methods:**

A retrospective analysis was performed on the clinical data of patients with POP stage III or above uterine prolapse treated at Wenzhou People's Hospital from January 2013 to December 2019. Patients were divided into two groups: USCLF group and SSLF group. The perioperative indicators, postoperative complications, pelvic organ prolapse quantification (POP-Q), Pelvic Floor Distress Inventory-20 (PFDI-20), and POP/Urinary Incontinence Sexual Questionnaire-12 (PISQ-12) scores of the groups were analyzed and compared.

**Results:**

(1) The operative time and intraoperative blood loss in the USCLF group were lower than those in the SSLF group, with statistical significance (*p* < 0.05). (2) The incidence of postoperative buttock pain in the SSLF group was 10.7% (6/56), higher than that in the USCLF group (0/56) (Fisher's exact test, *p* = 0.027). (3) At one year of follow-up, significant improvement in Aa, Ba, C, Ap, and Bp values was observed in both groups (*p* < 0.05). The values of the Aa and Ba sites in the USCLF group were lower than those in the SSLF group one year after surgery (*p* < 0.05). (4) The PFDI-20 and PISQ-12 scores of the groups one year after surgery were lower than those before surgery (*p* < 0.05).

**Conclusion:**

Uterosacral and cardinal ligament suture fixation leads to less bleeding and better postoperative quality of life than preoperative and may be better than SSLF at preventing the recurrence of anterior wall prolapse after surgery.

## 1. Introduction

Pelvic organ prolapse (POP) is a common pelvic floor disorder in middle-aged and older women. There are more than 100 surgical methods for the treatment of POP. Pelvic floor reconstruction surgery with mesh has limited applications due to mesh exposure, nerve or major vascular injuries, and groin/hip pain [1, 2]. In recent years, surgeons have tended to use the patient's own fascia and ligament tissue for pelvic floor repair. Sacrospinous ligament fixation (SSLF) was first reported by Sederl [3] in 1958 and was widely used because it required no mesh placement [4]. After over half a century of development, SSLF gradually became the mainstream surgical method for vaginal vault prolapse treatment [5]. However, the position of the sacrospinous ligament is deep and difficult to expose and operate on, which may lead to rectal and nerve injury risks and postoperative complications such as painful intercourse and pelvic pain [6].

DeLancey reported that the maintenance of normal pelvic floor anatomy depended on three levels of support with the vagina as the supporting axis. In Level I (apical support), the cervix and upper top of the vagina are suspended to the pelvic walls by the uterosacral ligament and cardinal ligament. In Level II (horizontal support), the middle third of the vagina was suspended laterally to fascial structures (the arcus tendineus fascia pelvis or fascial arch and a similar posterior structure), and in Level III (distal support), there exists the fusion of the vagina with surrounding structures such as the levator ani muscles and the perineal body [7]. Among the three levels, the uterosacral and cardinal ligament (USCL) at level I could play a key role in supporting the vaginal vault [8]. Based on years of experience with pelvic floor surgery, in vaginal vault prolapse, our hospital reconstructs the apex of the vagina using suture fixation for bilateral uterosacral and cardinal ligaments. In this study comparing the efficacy of uterosacral and cardinal ligament fixation (USCLF) and SSLF, the advantages and disadvantages of uterosacral and cardinal ligament suture fixation are comprehensively evaluated to increase the current understanding of pelvic floor reconstruction.

## 2. Materials and Methods

### 2.1. Selection and Assignment of Subjects

The patients with uterine prolapse treated in the Wenzhou People's Hospital from January 2013 to December 2019 were selected. Inclusion criteria include ① complete clinical data and ② quantitative staging of POP (POP-Q) [9] used for diagnosis, ranging from stages III to IV. Exclusion criteria include ① genitourinary tract infection; ② moderate-to-severe stress urinary incontinence (SUI) evaluated by physical examination and 1-hour urine pad test [10]; ③ gynecological neoplastic diseases; and ④ incomplete general information. The patients were divided into the USCLF group and the SSLF group according to different surgical methods. Those in the USCLF group were treated with transvaginal hysterectomy (TVH) combined with anterior and posterior vaginal wall repair combined with uterosacral and cardinal ligament fixation, while those in the SSLF group were treated with TVH combined with anterior and posterior vaginal wall repair combined with SSLF. This study was approved by the medical ethics committee of Wenzhou People's Hospital. This study was conducted in accordance with the Declaration of Helsinki. Written informed consent was obtained from all participants before surgery.

### 2.2. Preoperative Preparation

Information on the patients' detailed medical history and physical examination, gynecological examination, urine sediment examination with bacteriological analysis, and transvaginal ultrasound were assessed. Urinary incontinence was evaluated with a physical examination, urodynamics, and a 1-hour pad text to assess the type and severity of urinary incontinence. Surgical infection prevention included antibiotic administration 30 minutes before surgery.

### 2.3. Surgical Technique

Surgeries were performed by an associate chief physician or chief physician. All patients were treated with vaginal hysterectomy plus repair of the anterior and posterior vaginal walls as the basic surgery, and the SSLF group was further treated with SSLF. In the USCLF group, USCLF was performed in addition to the basic operation.

The SSLF is a mainstream surgical procedure for treating apical vaginal prolapses [11]. It is used to correct pelvic tissue defects. The sacrospinal ligament, with its constant position and strong strength, is an effective attachment point for vaginal suspension. Due to the low exposure of suture points, small operating space, and suturing difficulty, SSLF surgery often requires the use of special suture instruments, which has become an important factor limiting the promotion of SSLF in primary hospitals. To prevent postoperative apical vaginal prolapses, the USCL is fused, and the stump of the vagina is sutured in place during transvaginal hysterectomy. On this basis, our hospital made a procedural improvement by separating the bilateral USCL with a length of 2 cm from the broken end to the depth for overlapping and shortened sutures. A new USCL complex was formed using the cardinal ligament, uterine sacral ligament, and surrounding connective tissue, and the apex of the vagina was fixed to the complex so that the tip's anchor point reached the level of the ischial spine to reconstruct the first level better.

**Figure 1:**
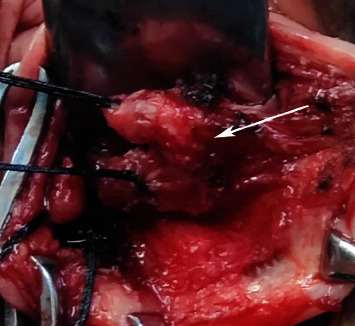
The left uterosacral-cardinal ligament stump was separated by 2 cm: arrow shows the stump of the uterosacral-cardinal ligament (↑).

**Figure 2:**
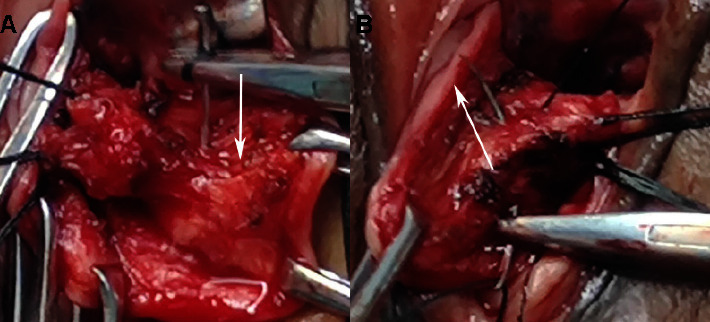
The U-shaped suturing of the bilateral uterosacral-cardinal ligament. (a) The left uterosacral-cardinal ligament needle entered (↓). (b) The right uterosacral-cardinal ligament needled out (↑).

**Figure 3:**
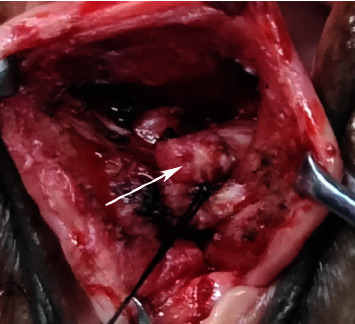
The uterosacral-cardinal ligament complex was formed (↑).

The surgical procedures of SSLF are referred to in the literature [12]. The procedure of the USCLF was as follows: ① After transvaginal hysterectomy, the pelvic peritoneum was closed and hemostasis was checked. ② The USCL complex was bluntly separated, and the stump was separated by 2.0 cm (Figure 1). ③ The one side of the USCL was sutured to the other side using a 1-0 nonabsorbable suture U-shaped suture and crossing overlap of the two sides of the USCL to form a new uterosacral-cardinal ligament complex (Figures 2 and 3). ④ The apex of the vagina was anchored to the USCL complex while the anterior vaginal wall was repaired.

### 2.4. Outcome Evaluation

All patients were followed up in the clinic for 3, 6, and 12 months postsurgery and once a year thereafter.

#### 2.4.1. Perioperative Outcomes

Operative time, intraoperative blood loss, length of hospital stay, and indwelling catheter time were analyzed and compared between groups. Visual estimation and a combination of gravimetry and direct measurement were used for assessing the blood loss. The urinary catheter was removed 3 days postsurgery, and perineal sutures were removed 7 days postsurgery before patients were discharged. Intraoperative and postoperative complications were recorded. Complications include infection, urinary retention, hematoma, and hip pain.

#### 2.4.2. Objective Evaluation of POP

The locations of each indicator point (i.e., Aa, Ba, C, Ap, and Bp), total vaginal length (TVL), and the vaginal diameter were measured before surgery, three months after surgery, and one year after surgery.

#### 2.4.3. Subjective Evaluation of POP

Quality of life was assessed using the Pelvic Floor Distress Inventory-20 (PFDI-20). The lower the score, the better the quality of life [13]. The assessment of sexual function was based on the POP/Urinary Incontinence Sexual Questionnaire-12 (PISQ-12) [14]. The higher the score, the better the sexual function. Both forms were completed before surgery and 12 months after surgery.

#### 2.4.4. Assessment of Recurrence

Recurrence was defined as the occurrence of following conditions: (1) the tip of the vagina descended more than one-third of the length of the vagina; (2) the anterior or posterior wall of the vagina descended to the hymen margin; (3) reported bothersome bulge symptoms by the participant in response to the questions, “Do you usually have a sensation of bulging or protrusion from the vaginal area?” or “Do you usually have a bulge or something falling out that you can see or feel in the vaginal area?” on the Pelvic Floor Disorders Inventory (PFDI); or (4) the participant received surgery or was elected to use a pessary for prolapse at any point during follow-up [15].

### 2.5. Statistical Analysis

The SPSS 13.0 software was used for the statistical analysis of the data. The mean/standard deviation and median/interquartilic range were used to describe variables with a normal and not normal distribution, respectively. Categorical variables were described as *n* (percentage). The independent sample *t*-test was used to compare continuous variables with a normal distribution or if the distribution was not normal, the Mann–Whitney *U* test was used instead. The chi-square test or Fisher's exact test was used to evaluate the association between nominal variables.

## 3. Results

A total of 112 patients with POP were included in this study, with 56 cases in each group. There was no significant difference in the clinical data between the groups (Table 1).

### 3.1. Comparison of Perioperative Clinical Indicators between the Two Groups

The operation time and intraoperative blood loss in the USCLF group were lower than those in the SSLF group (*t* = 4.405, *p* < 0.001; *t* = 2.902, *p*=0.004). There were no significant differences in the indwelling catheter time or hospital stay between the groups (*t* = 1.464, *p*=0.146; *t* = 1.713; *p*=0.089) (Table 2).

### 3.2. Comparison of Perioperative Complications between the Two Groups

Both groups survived the perioperative period safely, and no blood transfusion, local hematoma, ureteral injury (ureteral ligation/ureteral perforation), bladder injury, rectal injury, infection, or dysuria were recorded. Right buttock pain occurred in six patients in the SSLF group (10.7%, 6/56), while no buttock pain occurred in the USCLF group (Fisher's exact test, *p*=0.027).

### 3.3. Objective Evaluation of POP

The Aa, Ba, C, Ap, and Bp values in both groups at one year after surgery were all lower than those before surgery (Mann–Whitney *U*-test, *p* < 0.05).The Aa and Ba sites in the USCLF group were lower than those in the SSLF group at one year after surgery (Mann–Whitney *U*-test, *p*=0.011, *p*=0.007) (Table 3). After surgery, the vagina of all 112 patients could accommodate 2-3 fingers. In the SSLF group, the angle of the vaginal stump was skewed to the right, and the vaginal axis was tilted backward.

### 3.4. Subjective Efficacy of POP

According to the PFDI-20 score questionnaires, 105 in 112 (98.3%) cases were considered valid. There were 25 patients who were sexually inactive after surgery and 10 who were widowed. According to the PISQ-12 questionnaires, 77 cases were considered valid (68.8%). The PFDI-20 and PISQ-12 scores of the groups one year after surgery were lower than those before surgery (*t*-test, *p* < 0.001). There was no significant difference in the PFDI-20 or PISQ-12 score between groups (*t*-test, *p* > 0.05) (Table 4).

### 3.5. Assessment of Recurrence

All patients were followed up for more than two years after the operation, and any recurrence within this time was compared between the groups. Two patients in the SSLF group and two patients in the USCLF group had relapses. Four patients presented with anterior vaginal wall prolapse to 1 cm above the hymen six months to two years after the operation, accompanied by a slight feeling of the pelvic abdomen falling. However, the anterior vaginal wall prolapse did not progress, and none of the patients received reoperation or pessary treatment.

## 4. Discussion

### 4.1. Importance of Apical Vaginal Prolapse Repair

The supporting structures of the uterus and pelvic floor include three suspensory ligaments, pubourethral, cardinal/uterosacral, and arcus tendineus fascia pelvis [16]. Of these, the USCL complex is the cornerstone supporting the pelvic floor structure [17]. Damage to this complex can destroy the integrity of the septum, resulting in symptoms such as vaginal and uterine prolapse. Summers et al. [18] and Lowder et al. [19] demonstrated that 50% of anterior pelvic support comes from the apex of the vagina and that apical restoration can solve 55% of anterior vaginal wall prolapse and 33% of posterior vaginal wall prolapse. Clinical practice has shown that 30% to 50% of mild anterior and posterior vaginal wall prolapse no longer requires anterior and posterior vaginal wall repair after top reduction [20]. In a follow-up of 3,244 patients 10 years after POP, Eilber et al. [21] found that 20.2% of those who had only anterior vaginal wall repair underwent a second surgery for recurrence, while this rate was 11.6% in patients who underwent apical vaginal suspension on the basis of anterior vaginal wall repair. Therefore, the key point of pelvic floor reconstruction is the suspension of the top of the vagina.

### 4.2. Indications of TVH with Apical Vaginal Prolapse Repair

Some scholars opine that preventive apical vaginal reconstruction can be performed on patients who are at a high risk of apical vaginal prolapse after hysterectomy [4, 22]. SSLF or USCLF can be selected for patients with degree III or IV uterine prolapse, where the apex of the vagina may prolapse to or beyond the vaginal orifice under traction after hysterectomy; SSLF is recommended if the uterosacral ligament is found to be apparently lax or weak and cannot be used as support during the surgery [23].

### 4.3. Safety and Complications of SSLF and USCLF

The rectum, sciatic nerve, internal pudendal artery, pudendal nerve, and presacral vascular plexus surround the sacrospinous ligament. The uterosacral ligament (USL), 12–14 cm in length, includes the cervix/distal part, the middle part, and the sacrum/proximal part. The thickest distal part is about 2-3 cm in length and fuses with the cardinal ligament (CL) near the cervix and the superior vaginal segment. The superior gluteal veins and arteries are directly below the distal part of the USL, and the inferior rectal arteries are at the lower middle margin of the USL [24, 25]. The deep USL is rich in sympathetic nerves (sensory/pain fibers) with a small number of parasympathetic fibers [26].

CL is the connective tissue that encloses the blood vessels and the pelvic plexus from the internal iliac artery to the lateral margins of the cervix and vagina [27]. Chen et al. [28] found that CL is rich in nervous tissue, with the parasympathetic nerves dominating in the distal segment, followed by the middle segment, and then the proximal segment. During vaginal floor reconstruction, the distal part and middle part of the USL or CL are often used to fix the uterine and vaginal fornix. SSLF and USCLF have surgical complications such as bleeding and hematoma caused by adjacent vascular injury, hip, leg, perineal pain, or abnormal urination caused by nerve injury, and injury to and infection of the urinary catheter, bladder, and rectum.

In the two groups reported in this paper, there was no intraoperative or postoperative bleeding, organ injury, or postoperative infection. The urinary catheter was routinely removed 3 days postsurgery, and perineal sutures were removed 7 days postsurgery before patients were discharged; thus, the duration of the indwelling urinary catheter and length of hospital stay were longer than usual. The USCL can be separated and sutured under direct vision, making it easier to operate. The results of this study showed that the procedure results in a shorter operative time and less blood loss than SSLF. Postoperative buttock pain occurs in 10%–15% of patients with SSLF [29]. In this study, there were six patients with postoperative buttock pain in the SSLF group, with an incidence of 10.7%, while no postoperative pain was found in the USCLF group. This was due to the anatomy around the anchors at the top of the vagina. The region of the sacrospinous ligament without nerve distribution is only a third of the medial part of the sacrospinous ligament, and appropriate suture points must be selected to reduce the incidence of postoperative pain. The suture point of the USCLF is located about 2 cm from the cervical end of the cardinal ligament and the uterine sacral ligament. It should be noted that the ureter is near here, making it necessary to guard against ureteral injury, distortion, and obstruction. No such serious complications occurred in the USCLF group in this study. The operation of USCLF used blunt separation to separate the USCL and simultaneously push the ureter away to avoid angulation and folding of the ureter.

### 4.4. Therapeutic Effect of SSLF and USCLF on POP

The POP-Q indicators in both groups were significantly decreased after the surgery compared with those before the surgery. However, the statistics showed that the Aa and Ba values in the USCLF group were lower than those in the SSLF group one year after surgery. Many studies have reported that recurrent vaginal anterior wall prolapse is the most common complication of SSLF [30, 31]. Sacrospinous ligament fixation causes the vagina to be axially tilted to the suspended side and backward, increasing the risk of anterior pelvic defects [32]. The apical vaginal anchors of the new procedure are more anterior than those of SSLF, which explains the difference in Aa and Ba measurements between the groups in this study. However, according to the results, there was no statistically significant difference in the recurrence of anterior vaginal prolapse between the groups. Thus, large, long-term studies are needed to confirm whether the new procedure is more effective in preventing postoperative pelvic defects.

### 4.5. Effect of SSLF and USCLF on Symptom Improvement and Sexual Life of Patients with POP

Some studies have analyzed the prolapse symptoms and sexual life quality improvement of POP patients with different prolapse degrees who received hysterectomy and SSLF, and they have significantly improved [33]. The current study's results also showed that the PFDI-20 and PISQ-12 scores of the USCLF and SSLF groups were significantly improved after surgery compared with those before surgery. There was no significant difference in the scores between the groups after surgery, suggesting that both the USCLF and SSLF can improve the quality of life and sexual life of patients with POP.

1. In summary, the advantages of using the complex formed by the overlapping and shortened suture of the USCL to fix the top of the vagina are as follows: ① There is less intraoperative bleeding, surgery under direct vision, and no vaginal mucosa dissociation. ② The incidence of postoperative buttock pain is lower. ③ USCLF had a better effect on maintaining Aa and Ba points one year postsurgery when compared to SSLF. However, its superiority in preventing postoperative anterior vaginal wall prolapse needs to be confirmed by long-term follow-up studies with a larger sample size. SSLF, where the point at which the apex of the vagina is fixed, is deeper and needs to be performed by an experienced physician, while USCLF, with a more superficial fixed point, may be a more practical option in primary hospitals. It provides a new treatment for apical vaginal prolapse, is easier to operate than SSLF, and is worthy of promotion in primary hospitals. The study also had some limitations. First, the study was retrospective, and patients were not randomly assigned to the study group and were assigned to the study group according to the surgeon's expertise, which may have biased the results. In addition, due to the limited number of cases and short follow-up time in this study, more advantages and complications in the long term after surgery have not been fully discovered. In the later period, it is still necessary to expand the sample size and conduct a longer and more multifaceted follow-up to observe the long-term effect.

## Figures and Tables

**Table 1:** Comparison of the clinical data between the two groups.

Groups	SSLF	USCLF	*p*
Age (year)	60.9 ± 7.1	63.5 ± 8.2	0.075^a^
Parity (times)	3.1 ± 0.8	3.1 ± 0.9	0.801^a^
BMI (kg/m^2^)	26.1 ± 1.6	25.7 ± 1.5	0.162^a^
Comorbidities (*n* (%))			
Uterine fibroids	18 (32.1)	20 (35.7)	0.701^b^
Hypertension	30 (53.6)	21 (37.5)	0.088^b^
Diabetes	12 (21.4)	8 (14.3)	0.450^b^
Urinary incontinence	5 (8.9)	7 (12.5)	0.541^b^
Anemia	8 (14.3)	13 (23.2)	0.262^b^
Chronic bronchitis	8 (14.3)	5 (8.9)	0.339^b^
Cerebrovascular disease	5 (8.9)	3 (5.4)	0.463^b^

Notes: The date of age, parity, and BMI are mean ± SD. The date of complications are *n* (%). ^a^*t* test. ^b^*χ*^2^ test.

**Table 2:** Comparison of perioperative indicators between two groups (mean ± SD).

Groups	*n*	The operation time (min)	Intraoperative bleeding (ml)	Hospitalizatio*n* time (d)	Time of indwelling catheter (d)
USCLF group	56	118.6 ± 18.6	102.7 ± 24.6	7.6 ± 1.3	3.3 ± 1.0
SSLF group	56	136.7 ± 24.5	120.0 ± 37.8	8.2 ± 2.0	3.8 ± 2.1
*T*		4.405	2.902	1.713	1.464
*p*		<0.001	0.004	0.089	0.146

**Table 3:** Comparison of POP-Q indicators between two groups (M (P25, P75)).

Indication points	Time	USCLF group	SSLF group	*Z*	*p*
Aa	Preoperation	2 (0, 2)	2 (1, 3)	−1.138	0.255
	One year after operation	−3 (−3, −3)	−3 (−3, −2)	−2.541	0.011
*Z*		−9.447	−9.204		
*p*		<0.001	<0.001		

Ba	Preoperation	3 (2, 4.5)	3 (2, 5)	−1.089	0.276
	One year after operation	−3 (−3, −3)	−3 (−3, −2)	−2.678	0.007
*Z*		−9.380	−9.017		
*p*		<0.001	<0.001		

C	Preoperation	2 (2, 4)	3.5 (2.5, 5)	−1.043	0.297
	One year after operation	−7 (−8, −6)	−7 (−7, −6)	−1.984	0.051
*Z*		−9.329	−9.256		
*p*		<0.001	<0.001		

Ap	Preoperation	0 (−1, 1)	0 (−1.875, 2)	−0.664	0.507
	One year after operation	−3 (−3, −3)	−3 (−3, −2)	−0.233	0.816
*Z*		−8.873	−8.881		
*p*		<0.001	<0.001		

Bp	Preoperation	0 (−0.75, 1)	0 (−1.375, 2)	−0.701	0.483
	One year after operation	−3 (−3, −3)	−3 (−3, −3)	−0.434	0.664
*Z*		−9.081	−8.647		
*p*		<0.001	<0.001		

TVL	Preoperation	8 (7, 10)	8 (7, 9)	−1.388	0.165
	One year after operation	8 (7, 9)	7 (7, 9)	−1.228	0.220
*Z*		−0.572	−0.554		
*p*		0.568	0.579		

Notes: Data are median (P25, P75). POP-Q = pelvic organ prolapse quantification system. Aa = a point located in the midline of the anterior vaginal wall 3 cm proximal to the hymen. Ba = the most dependent part of the anterior vaginal wall. C = the most dependent part of the cervix or the vaginal cuff if patient has no cervix. Ap = A point located in the midline of the posterior vaginal wall 3 cm proximal to the hymen. Bp = the most dependent part of the posterior vaginal wall. TVL = total vaginal length.

**Table 4:** Comparison of PFDI-20 and PISQ-12 scores between the study group and the SSLF group before and after treatment (score, (mean ± SD)).

Groups	PFDI-20 score					PISQ-12 score				
	*n*	Preoperation	One year after operation	*t*	*p*	*n*	Preoperation	One year after operation	*t*	*p*
USCLF group	52	73.9 ± 19.0	11.7 ± 7.5	22.777	<0.001	41	15.8 ± 5.6	34.8 ± 8.1	−14.422	<0.001
SSLF group	53	79.8 ± 21.0	14.4 ± 8.8	21.579	<0.001	36	17.4 ± 6.4	32.0 ± 8.3	−10.480	<0.001
*T*		−1.563	−1.738				−1.340	1.776		
*p*		0.121	0.085				0.183	0.078		

Notes: PFDI-20 = Pelvic Floor Distress Inventory-20. PISQ-12 = POP/Urinary Incontinence Sexual Questionnaire-12.

## Data Availability

The datasets used and/or analyzed during the current study are available from the corresponding author upon reasonable request.

## Abbreviations

POP: Pelvic organ prolapse
SSLF: Sacrospinous ligament fixation
POP-Q: Pelvic organ prolapse quantification
BMI: Body mass index
PISQ-12: Pelvic Organ Prolapse/Urinary Incontinence Sexual Questionnaire-12
USCL: Uterosacral and cardinal ligament.
